# The Use of Technology in Identifying Hospital Malnutrition: Scoping Review

**DOI:** 10.2196/medinform.7601

**Published:** 2018-01-19

**Authors:** Dino Trtovac, Joon Lee

**Affiliations:** ^1^ Health Data Science Lab School of Public Health and Health Systems University of Waterloo Waterloo, ON Canada

**Keywords:** hospital malnutrition, technology-driven health care, malnutrition detection, nutrition diagnosis, malnutrition assessment, food-intake monitoring, automated data, malnutrition, nutritional status, nutrition assessment

## Abstract

**Background:**

Malnutrition is a condition most commonly arising from the inadequate consumption of nutrients necessary to maintain physiological health and is associated with the development of cardiovascular disease, osteoporosis, and sarcopenia. Malnutrition occurring in the hospital setting is caused by insufficient monitoring, identification, and assessment efforts. Furthermore, the ability of health care workers to identify and recognize malnourished patients is suboptimal. Therefore, interventions focusing on the identification and treatment of malnutrition are valuable, as they reduce the risks and rates of malnutrition within hospitals. Technology may be a particularly useful ally in identifying malnutrition due to scalability, timeliness, and effectiveness. In an effort to explore the issue, this scoping review synthesized the availability of technological tools to detect and identify hospital malnutrition.

**Objective:**

Our objective was to conduct a scoping review of the different forms of technology used in addressing malnutrition among adults admitted to hospital to (1) identify the extent of the published literature on this topic, (2) describe key findings, and (3) identify outcomes.

**Methods:**

We designed and implemented a search strategy in 3 databases (PubMed, Scopus, and CINAHL). We completed a descriptive numerical summary and analyzed study characteristics. One reviewer independently extracted data from the databases.

**Results:**

We retrieved and reviewed a total of 21 articles. We categorized articles by the computerized tool or app type: malnutrition assessment (n=15), food intake monitoring (n=5), or both (n=1). Within those categories, we subcategorized the different technologies as either hardware (n=4), software (n=13), or both (n=4). An additional subcategory under software was cloud-based apps (n=1). Malnutrition in the acute hospital setting was largely an unrecognized problem, owing to insufficient monitoring, identification, and initial assessments of identifying both patients who are already malnourished and those who are at risk of malnourishment. Studies went on to examine the effectiveness of health care workers (nurses and doctors) with a knowledge base focused on clinical care and their ability to accurately and consistently identify malnourished geriatric patients within that setting.

**Conclusions:**

Most articles reported effectiveness in accurately increasing malnutrition detection and awareness. Computerized tools and apps may also help reduce health care workers’ workload and time spent assessing patients for malnutrition. Hospitals may also benefit from implementing malnutrition technology through observing decreased length of stay, along with decreased foregone costs related to missing malnutrition diagnoses. It is beneficial to study the impact of these technologies to examine possible areas of improvement. A future systematic review would further contribute to the evidence and effectiveness of the use of technologies in assessing and monitoring hospital malnutrition.

## Introduction

An inadequate diet can cause malnutrition, a condition where an individual is not consuming sufficient amounts of nutrients necessary to maintain their current level of physiological health [1].

Malnutrition increases the risk of developing certain chronic diseases, such as cardiovascular disease, osteoporosis (a debilitating loss of bone density), and sarcopenia (a debilitating loss of muscle tissue, mass, and function) [1,2]. Additionally, in hospitalized patients, malnutrition is associated with a diverse range of unfavorable outcomes, such as lowered immune function, higher infection rates, higher surgical complication rates, increased muscle loss, impaired wound healing, and overall increased morbidity and mortality [2].

Hospital malnutrition has been a historical problem as well as a current troubling issue, with documented prevalence rates as high as 60% in some hospitals [2]. Malnutrition in the acute hospital setting is suggested to be an unrecognized problem, owing to insufficient monitoring, identification, and initial assessments of patients who are already malnourished and those who are at risk [3-6]. Results suggest that health care workers’ ability to accurately and consistently identify malnourished geriatric patients is suboptimal in recognizing and monitoring malnourished patients and those at risk of malnutrition [3].

Addressing, identifying, and monitoring malnutrition properly may improve the outlook for older hospitalized patients. Interventions that focus on identification and treatment of malnutrition have positive influences on decreasing the risks and rates of malnutrition in hospitals [7]. Furthermore, there is a demand for enhanced nutrition monitoring and for standardizing food intake processes early and systematically [8-10]. Other studies have found a need for development and innovation in the area of nutrition informatics [11,12]. With the current trend of technological advancement, implementation of electronic health records, and the evolving use of health data, the field is positioned to advance best practices of nutrition care to primary care settings and specifically for patients [13,14]. Consequently, how and what types of technologies are being used in this field needs to be understood in depth.

There are no known scoping reviews, to the best of our knowledge, that have synthesized the availability of technological tools to detect and identify hospital malnutrition. Therefore, we undertook a scoping review of the literature to identify the extent of the published literature on this topic, describe key findings, and identify outcomes.

## Methods

We followed Arksey and O’Malley’s [15] scoping review methodological framework (which is generally recognized as best practice for scoping reviews), with the following stages.

### Development of Research Questions

The research questions were as follows: What is the extent of the published literature on using technology to monitor and assess malnutrition within the hospital setting? What is known from the existing literature about the impact and outcomes of implementing technology in such a manner?

### Search Strategy Development

We initially developed the complete search in PubMed (Textbox 1) and then adapted it for the Scopus and CINAHL databases.

Multimedia Appendix 1 shows the search terms used in the search strategies in PubMed, Scopus, and CINAHL. The searches were conducted between August 9 and 15, 2017. Following the search of the 3 databases and article selection, we also reviewed references in the included studies to ensure that we considered all possible relevant articles.

### Selection Criteria

We selected primary articles for inclusion in the scoping review if they discussed applicable technology-based approaches to monitor or assess malnutrition within the hospital or primary care setting. Articles not written in or translated into English were not included.

The definition of “applicable” included systems that were described, designed, or in current use and application at the hospital or primary care setting. We screened articles to determine relevancy of the systems.

**Keyword search strategy for PubMed (MeSH: Medical Subject Heading; tw: text word).**

(Nutrition [tw] OR Malnutrition [tw] OR Nutritional [tw] OR Hospital malnutrition [tw] OR Dietary assessment [tw] OR Food habits [tw] OR Eating [tw] OR Diet records [tw] OR Nutritional assessment [tw] OR nutrition support [tw] OR food habits [MeSH] OR eating [MeSH] OR diet records [MeSH] OR nutritional assessment [MeSH])

AND

(Monitoring [tw] OR Screening [tw] OR Technology-based dietary assessment [tw] OR Food record [tw] OR Recording [tw] OR Assessment [tw])

AND

(Device [tw] OR informatics [tw] OR technology [tw] OR computer [tw] OR Web based [tw] OR image based [tw] OR image retrieval [tw] OR picture [tw] OR digital photography [tw] OR mobile device [tw] OR mobile technology [tw] OR smartphone [tw] OR technology assist [tw] OR multimedia tool [tw] OR electronic [tw] OR wearable [tw] OR signal processing, Computer-Assisted/instrumentation* [MeSH] OR software [MeSH] OR wireless technology/instrumentation* [MeSH])

AND

(hospital [tw] OR primary care [tw] OR care [tw])

Technology-based approaches referred to any tool or system that was technology driven or computer based, and that comprised a hardware component or a software component (including Web- and cloud-based apps). Essentially, technology-based approaches excluded any conventional nutrition support tools that only included paper-based methods traditionally used for nutrition assessment.

We excluded studies that primarily considered nontechnologically driven or noncomputer-based solutions to address hospital malnutrition. We also excluded technologically driven or computer-based solutions based in hospitals, such as hospital accounting systems or inventory systems, that had no relevance to nutrition. For the purposes of our review, we excluded studies that primarily dealt with nutrition management systems focusing on prenatal and neonatal patients. The primary aim of this scoping review was to identify technologically relevant systems for use in hospitals for adults.

### Article Selection and Data Extraction

We selected articles in the following 2 phases: (1) title, abstract, and keyword review phase, and (2) full-text review phase.

Phase 1 review considered all search results. We developed a relevance form adapted from the template used by Griebel et al [16] to aid the phase 1 review (Multimedia Appendix 1). In the phase 2 review, we included articles that met the inclusion criteria from the phase 1 review. We screened and subsequently removed duplicate articles. Titles for which an abstract was not available were included for phase 2 review if the title was enough to indicate that technologically driven nutrition management tools or systems were being discussed. For those that we deemed relevant after phase 1 and 2 reviews, we obtained the full-text articles and included them in this scoping review.

### Data Charting

We organized the articles according to what the tool or app measured: malnutrition assessment, food intake monitoring, or both. Within these 3 categories, we subcategorized the articles by the type of technology used: software (including cloud-based apps), hardware based (including portable devices), or both.

### Collation and Summary of the Results

The goal of this scoping review was to analyze eligible articles to obtain an overview of the scientific literature on technological and computer-based approaches to nutrition monitoring and assessment in hospitals. With this goal in mind, we summarized and present the collection of key messages and concepts from eligible publications. We developed a data synthesis and characterization form to include the following study characteristics: authors; year of publication; country of origin; publication type; aims and purpose; description of the patients or participants; description of the article; type of technology used (hardware, software, or both); availability of the tool or app (theoretic, prototypic, in use, or validated); outcomes of the intervention; and cost implications (if available).

## Results

### Descriptive Summary

**Figure 1:**
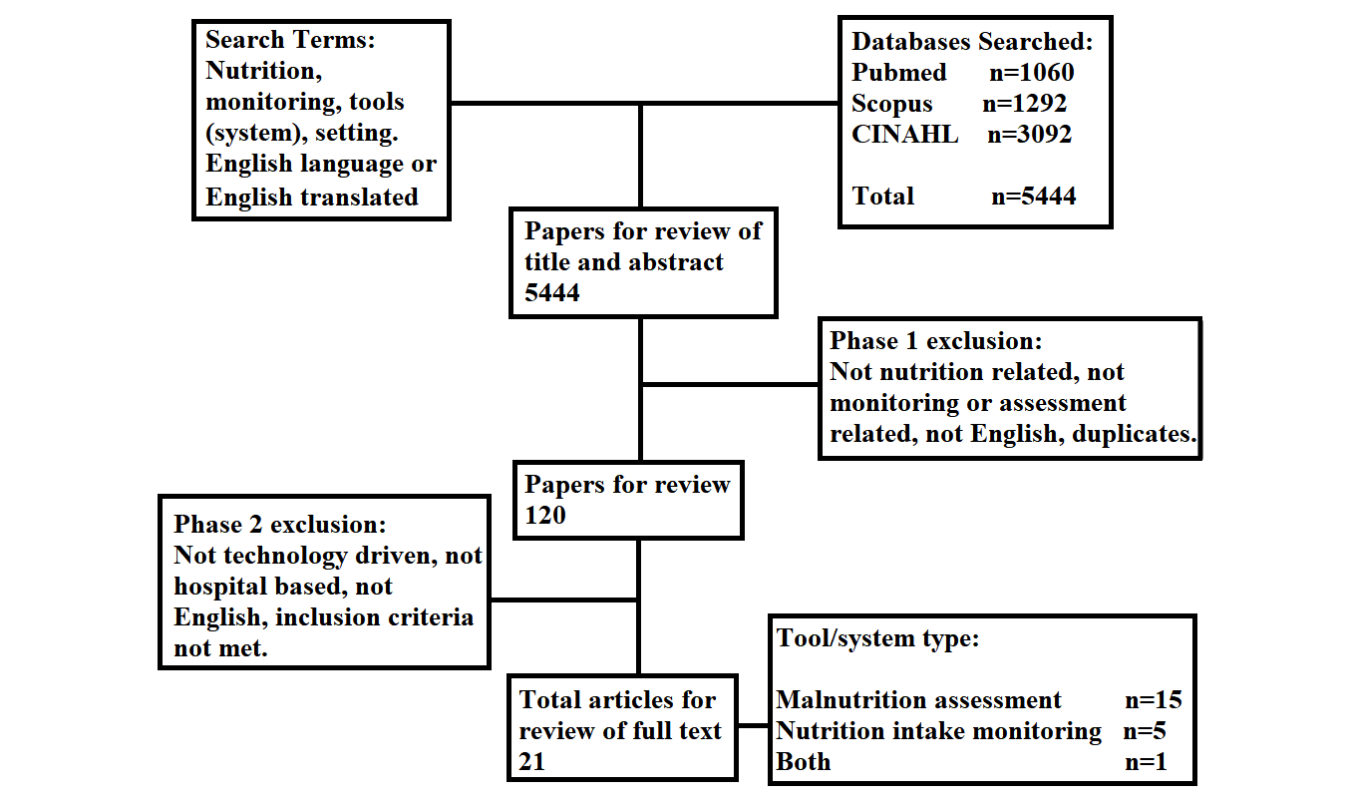
Flowchart summarizing the results.

The search returned 5444 articles. We deemed a total of 21 of those articles to be relevant after phase 1 and 2 reviews. Malnutrition assessment was the most frequent aim (n=15, 71%), followed by nutrition intake monitoring (n=5, 24%), followed by an app that measured both components (n=1, 5%) (Figure 1).

Most of the malnutrition assessment articles discussed technology based on software created and incorporated within the hospital’s own computer system (n=12, 80%). Subcategorized within the software category was an article that proposed a cloud-based system for malnutrition assessment (n=1, 7%). Some studies directly used a hardware component (portable device) that assessed malnutrition (n=2, 13%).

Among the food intake monitoring articles, 2 (40%) studies contained a hardware component and 3 (60%) used technology that contained both a hardware and a software component.

**Figure 2:**
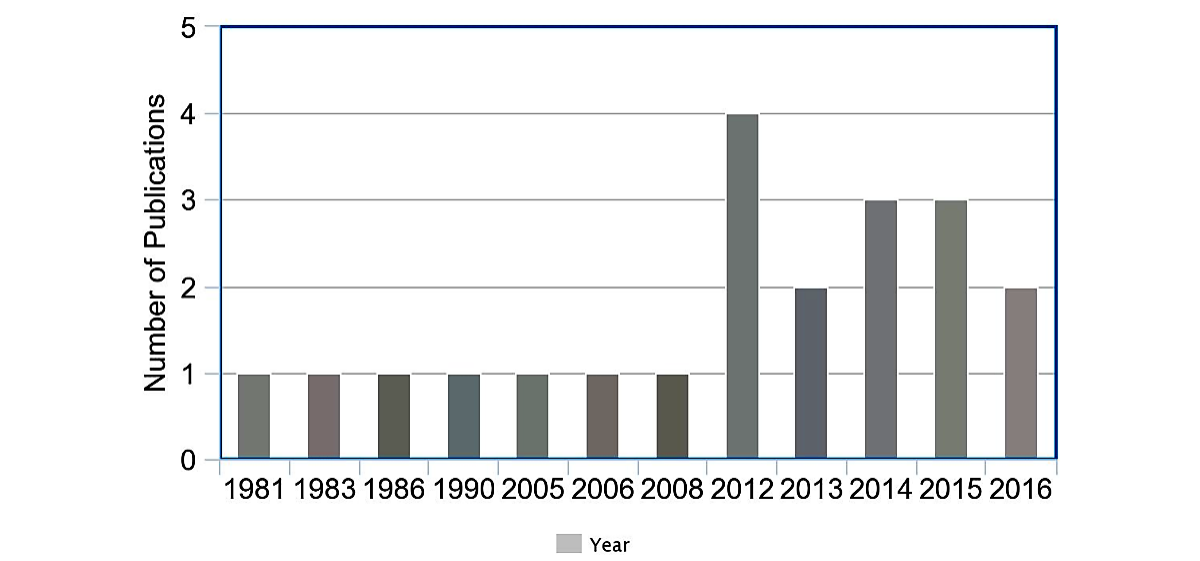
Number of articles by year of publication.

One article mentioned an inclusive nutrition system that contained technology for both malnutrition assessment and food intake monitoring. Multimedia Appendix 2 details all of the primary articles that we retrieved and characterized for this review. Most of the articles were published from 2012 to 2016 (Figure 2).

The largest share of publications on the topic originated from the United States (n=7, 33%), followed by the United Kingdom (n=4, 19%), Spain (n=2, 10%), Taiwan, Philippines, the Netherlands, France, China, Australia, Argentina, and Israel, each with 1 article (n=1, 4.8%).

Most of the articles reported semiexperimental studies (n=16), followed by descriptive studies (n=3), a randomized controlled trial (RCT; n=1), and a retrospective study (n=1). Semiexperimental studies may dominate the field because they all introduced a novel malnutrition assessment or nutrition intake monitoring tool, which was primarily being tested for accuracy and effectiveness (on patients, participants, or extracted data) against universally accepted and standardized malnutrition assessment tools (eg, Mini Nutritional Assessment-Short Form [MNA-SF], Subjective Global Assessment [SGA], or Full Nutrition Assessment [FNA]) or expert opinions.

Most of the studies tested their tools and apps on patients or participants (n=14). Additionally, most of the studies reported that their tool or app was able to increase malnutrition detection or awareness (n=14). The same articles reported a degree of accuracy respective to their tool or app as determined by using validated tools (eg, MNA-SF, SGA, or FNA) or clinical nutrition expert and dietician consultations or testing (n=15).

### Study Characteristic Analysis

#### Technological Interventions

A key concept mentioned in some of the articles that included a software component is automated data collection. For example, some software systems included features that allowed other departments within the hospital, such as the biochemical laboratory, to update a patient’s blood test results automatically [17-19]. In addition to data obtained from laboratory results, bioelectric impedance results were automatically updated and used to assess malnourishment [20-22]. (Bioelectric impedance is a noninvasive way to measure body fluid composition using electrodes that are placed on the wrist and ankle. The results of the measurement can be used as a parameter—in conjunction with other parameters, such as height and weight—to assess whether an individual is malnourished.) The corresponding data were then added into the patient’s file on the computer app, which in conjunction with biochemical markers could be used to accurately assess malnutrition.

Another feature of software apps was the ability to display a nutrition diagnosis (eg, undernourished, malnourished, low protein energy metabolism), an automated warning, or an alert once relevant data parameters were assessed. Some apps provided automated alerts that informed health care workers when a patient was assessed as being at risk for malnutrition [12,17,19,21]. Other software apps provided a malnutrition assessment when prompted, which was done by manual data entry of relevant parameters [23-25].

Only 1 study had cloud-based technological components. It allowed health care workers to create an account, log in, and begin manually entering patient data, such as biochemical markers [26]. Afterward, the system provided a corresponding nutrition diagnosis (underweight, malnourished, and 48 other diagnoses).

For the hardware component, portable devices were used to assess malnutrition risk. Largely based in the 1980s, these portable devices were pocket computers, where physical, anthropometric, and laboratory results could be manually entered to produce a malnutrition assessment [27,28]. Included in the hardware component was a wearable device, eButton, designed to record real-time food intake and to assess the malnutrition risk via direct monitoring [29]. Another study discussed the use of hardware components (an electronic weight scale, a height scale, and a bioelectric impedance spectroscopy device) that, in conjunction with a computer program, could predict a patient’s likelihood of becoming malnourished [30]. Other articles discussing systems that encompassed both hardware and software components mentioned the use of a tablet that contained a “wipe-away” feature for monitoring food intake [10,30]. This concept enabled more accurate food estimations. It allowed health practitioners to wipe away corresponding food items on a tablet depiction of the food a patient was served. The software component could accurately estimate the amount of food eaten by the corresponding amount that had been erased.

#### Overall Outcomes

Most of the interventions had successful outcomes (n=16, 76%) [12,17-23,25-27,29,31-34]. One outcome that was mentioned in several of the articles was an improvement in time efficiency, which was facilitated by the technological implementation of the process [19,23,25-27,31,33,34]. Other articles reported that implementing a technological component improved detection rates of malnutrition within the hospital [12,17,20,22,28, 30,31,34]. Earlier detection allowed for a faster response to those patients with a diagnosis of malnutrition.

In addition to saving time and improving detection rates, 4 articles discussed the economic benefits and potential costs saved from incorporating the mentioned technology into practice [19,23,31,34]. Giovannelli et al [31] discussed how an automated email alert system for readmitted patients with previously known malnutrition decreased hospital length of stay (LOS). Rossi et al [19] expected their computer program to save an annual Aus $6500 to $10,000 in hospital costs related to malnutrition. McGurk et al [23] argued that their unique self-screening malnutrition assessment process would have the potential to reduce the workload of health care workers, thereby potentially cutting costs. Hershkovich et al [34], discussed how their hospital experienced funding cutbacks that led to a downsizing in the clinical nutrition department, which gave way to the development of the Rambam Automated Nutrition Computerized Screening (RANCS) tool. They argued that RANCS is a cost-effective option when downsizing in the clinical nutrition department is an issue.

#### Availability

Of the tools and apps reviewed, less than half (n=9) were available for further clinical testing and implementation within other hospital systems [17,20,23,25,27,30,31,33,34]. The remaining tools or apps were in the prototype or further testing stage [18,21,22,24,26,29,35] or were a theoretical concept [10,28], or no information was available on the tool’s or app’s availability [12,19,32].

## Discussion

The 2 research questions formulated at the beginning of this scoping review were as follows: What is the extensiveness of the existing literature? What are the key impacts and outcomes? We discuss the outcomes in terms of effectiveness and accuracy of tools and apps; financial implications; efficiency and educational implications; and future and ethical implications.

### Effectiveness and Accuracy of Tools and Apps

A total of 15 of the 21 tools and apps examined in this scoping review were effective in accurately increasing malnutrition detection and awareness using either malnutrition assessment apps or nutrition intake monitoring tools [12,19-23,26,27, 29,31-34,36].

Effectiveness was tested in a variety of ways, including evaluation studies, an RCT, and prototype testing conducted on a sample of patients or participants. The malnutrition assessment apps’ effectiveness and accuracy were tested against common practice-validated nutrition screening tools used globally by hospitals, such as the MNA-SF, SGA, and FNA tools; or they were directly assessed based on expert opinions of clinical dieticians and clinical nutrition experts. The nutrition intake monitoring tools’ accuracy and effectiveness were tested by comparing results from the estimated amount of food consumed with the actual amount of food consumed [29,33,36]. These results suggest that the applicability of the technological tools and apps may not *only* be effective, but they may also have a degree of accuracy similar to current and widely used conventional malnutrition assessment tools. However, only Brieux et al [12] conducted an RCT that directly assessed the effectiveness of using a software system to identify cases of malnutrition by comparing it with conventional paper-based methods. Therefore, reported improvements in accuracy and effectiveness over conventional methods from the other articles are limited by the degree of evidence due to study design methodology.

The results show that malnutrition assessment tools and apps were the dominant forms of technology used to measure malnutrition outcomes, while nutrition intake monitoring tools were used to a lesser extent. Malnutrition assessment tools and apps may dominate the literature because they may offer greater incentives and benefits than nutrition intake monitoring tools, which by their nature may require additional hospital expenditures on infrastructure (such as recording hardware or cameras). One possible incentive for using malnutrition assessment tools and apps may be their relative ease of implementation compared with nutrition intake monitoring tools. This may be due to the fact that most malnutrition assessment apps incorporated software programs, which may easily be adapted into a hospital’s existing electronic infrastructure [12,17,19-23,26,31,32,34].

Some of the most simplified apps used Excel files that were tied into an algorithm developed by the authors [23,31]. McGurk et al [23] described and tested their self-screening tool that allowed all hospital inpatients to measure and enter their own anthropometric parameters (weight and height) into the Excel file. This was designed to ensure that all possible patients would be screened for malnutrition. Favorably, the results showed that there was no significant difference or errors in measurement and data entry between health care workers and patients who self-screened. Giovannelli et al [31] described an Excel spreadsheet containing a list of all previous patients who were identified as being malnourished or at risk, and another Excel spreadsheet containing a list of all inpatients admitted within the last 24 hours. The authors developed an algorithm that automatically cross-referenced the 2 spreadsheets to potentially identify a matching name that appeared on both, which automatically alerted the nutrition department in the hospital. The effort required to introduce and build new hardware components may seem discouraging; however, studies such as the latter two may seem relatively appealing to hospitals should they chose to adopt and develop similar apps. In this scoping review, it was not possible to compare the effectiveness of malnutrition assessment apps versus nutrition intake monitoring apps because the studies’ sizes differed and because they measured different parameters.

### Financial Implications

From an economic perspective, malnutrition is associated with increased LOS and readmission rates within hospitals [35]. Paradoxically, prolonged LOS is associated with further decline in nutritional status, a sort of malicious cycle that occurs between the two elements [8]. Therefore, a decrease in the incidence of malnutrition may decrease the economic burden incurred by hospitals. Correia and Waitzberg [37] compared and evaluated hospital-related costs between malnourished patients and nourished patients; they found that hospital-related costs were 3 times as high for the malnourished patients. Similarly, other studies had comparable findings that malnourished patients had higher hospital-related costs than did nourished patients; even patients who were at risk of malnutrition had higher associated hospital-related costs [38-40].

A total of 3 of the reviewed articles mentioned economic incentives associated with implementing technology-based approaches for malnutrition assessment [19,23,31]. Although there was no direct monetary transaction, authors argued that economic incentives were in the form of reduced future hospital costs related to malnutrition. Such savings were argued to come from reduced health care worker hours through improved computer-assisted efficiency in processes and decreased LOS experienced by patients [19,23,31].

As health care costs in North America are on the rise and since the costs relating to malnutrition account for some of that expenditure, the results suggest that implementing such technology in the hospital setting could save costs [41]. Additionally, this type of cost savings may be appealing to hospitals because it differs from cost-cutting efforts that rely on laying off health care workers.

Because older patients experience increased rates of deconditioning within the hospital, hospitals may find it beneficial to undertake efforts that identify malnutrition faster than conventional methods. Giovannelli et al [31] suggested that detecting malnutrition sooner may elicit a faster response, thereby possibly reducing hospital LOS. Furthermore, their software program showed decreased rates of LOS and improved financial impacts on the hospital’s budget. Therefore, such technology may be of interest to hospitals, as it may mitigate the negative consequences associated with deconditioning and increased LOS.

The economic incentive is also extended to patients. They benefit not only physiologically from earlier malnutrition detection but also financially through decreased spending related to deconditioning outside of the hospital for homecare or nursing care [42].

In hospitals and other medical settings where there is a “pay-for-performance” infrastructure, the detection of malnutrition may result in *significantly* greater financial reimbursements. Gout et al [5] brought together multiple cases from hospitals in different countries that implemented a pay-for-performance infrastructure and described the foregone costs related to undetected malnutrition: 2 Australian studies reported an annual financial loss of Aus $1,850,540 and Aus $1,677,235, respectively, relating directly to undiagnosed and undocumented malnutrition [5]; 1 German study reported a €35,280 financial loss due to unrecognized and unidentified malnutrition [43]; and 1 US study reported a US $86,000 financial loss for a hospital in 1 year [44]. The evidence suggests that the implementation of such technology may add to the potential of hospitals to recoup any forgone revenues or reimbursements relating to unidentified cases of malnutrition.

### Efficiency and Educational Implications

The results suggest that technological nutrition support tools and apps may help reduce health care workers’ workload and time spent assessing patients for malnutrition [19,23,27,32-34]. This may be beneficial because these technologies can facilitate malnutrition assessment, monitoring, and awareness efforts with a smaller health care workforce or by encouraging the patients themselves to self-assess for malnutrition. Technological nutrition support tools and apps may also have an edge on conventional hospital malnutrition monitoring methods. Some of the studies showed that technological nutrition support tools and apps had the potential to improve the hospitals’ efficiency and effectiveness in identifying malnutrition [19]; detect *more* cases of malnutrition [12,34]; and improve the accuracy of clinical nutritional diagnoses [26].

The results also showed that certain tools and apps can collect relevant data either automatically or by being entered manually into the system. In our review, more articles discussed tools or apps that were able to automatically collect data. Some of the studies described their nutrition support technologies as being able to monitor, assess, and identify hospital malnutrition cases relatively independently or with minimal input from health care workers [12,17,19-22,29,31,33].

Technology that has the ability to run independently or with minimal input may be beneficial in hospital areas where noise pollution is abundant, or in areas where there is relatively little time for health care workers to manually collect and analyze malnutrition information (eg, in the intensive or critical care unit). Additionally, some hospitals choose to lay off health care workers, which increases the workload and patient to nurse ratio, adding further strain on malnutrition monitoring and assessment efforts [45]. Therefore, technology that can function independently may be beneficial in these areas to mitigate the negative associations relating to an increase in patient to nurse ratios.

Furthermore, nutrition support tools and apps may have educational implications for health care workers, including nurses and doctors. Dieticians and clinical nutrition experts have more years of formal schooling and experience relating to malnutrition assessment and identification than do nurses and doctors [46]. Nutrition education has a small role in medical schools and nursing schools and is further shown to be inadequate [46]. Few tools and resources are made available to nurses or doctors to expand their practical knowledge in this area. Therefore, tools and apps that have the ability to assess and monitor patient malnutrition with a user-friendly interface may be beneficial to these health care workers. All of the articles we reviewed tested or described a nutrition support tool or app that could provide a warning, alert, risk, or predictive outcome based on its respective form of data entry. These tools may have the potential to operate in a way that’s analogous to using a calculator to solve a complex math problem, where extensive nutrition knowledge is not necessary.

However, none of the articles discussed the ease of use from the user’s perspective. The importance of establishing a usability rating may help determine the tool’s or app’s *actual* usefulness in practical settings.

### Future and Ethical Implications

It is common practice that scoping reviews do not address issues of quality appraisal relating to the sources of literature used. In this scoping review, we tried to use and capture peer-based research articles that directly tested their tool or app on a cohort of patients or participants. A few articles in this scoping review contained technology in the *theoretical concept* stage [10,28]. As such, their findings are used to outline possible future directions of research within that respective domain.

Additionally, each of the reviewed research articles included different and unique types of software and hardware technologies respective to their study. Future research should seek to test the efficacy of one type of computer tool or app across different hospitals in the form of RCTs in order to contribute to a higher level of evidence for the field. It would also be interesting for future efforts to compare and test the outcomes of using malnutrition assessment tools and apps versus nutrition intake monitoring tools.

### Limitations

It is likely that we excluded some relevant research articles from this review, as we screened only English-language publications. Some studies (n=8) did not pass phase 2 relevance screening because of the language restriction. These studies might have added further applications and outcomes in this scoping review. Perhaps future efforts to translate these publications will add value and knowledge to the field. Lastly, because this was a review study, we based our analysis solely on the information found in each included article, which was up-to-date only at the time of publication.

### Conclusion

The use of technologies for monitoring food intake and assessing malnutrition are beginning to be considered for future hospital processes that aim to identify, diagnose, and assess hospital malnutrition. Many computerized tools and apps are being developed worldwide to address the problem of hospital malnutrition. It is beneficial to study the impact of these technologies to examine their applicability and possible areas of improvement.

## Appendix

Multimedia Appendix 1 Search terms by database.

Multimedia Appendix 2 Adapted data characterization form.

## Abbreviations

FNA: Full Nutrition Assessment
LOS: length of stay
MNA-SF: Mini Nutritional Assessment-Short Form
RANCS: Rambam Automated Nutrition Computerized Screening
RCT: randomized controlled trial
SGA: Subjective Global Assessment
