# Supplementary material for: The Use of Technology in Identifying Hospital Malnutrition: Scoping Review
Source: JMIR Med Inform. 2018 Jan 19;6(1):e4. doi: 10.2196/medinform.7601 (PMC5797288; doi:10.2196/medinform.7601)
Supplement: Multimedia Appendix 1 [file medinform_v6i1e4_app1.pdf]

APPENDICES

Appendix 1: Search Terms by Database

Table 1. Search terms used for PubMed Search Strategy

Concepts	Nutrition	Monitoring	Tools (systems)	Setting
**Author keywords**	• Nutrition<br>• Malnutrition<br>• Nutritional<br>• Hospital malnutrition<br>• Dietary assessment<br>• Food habits<br>• Eating<br>• Diet records<br>• Nutritional assessment<br>• Nutrition support	• Monitoring<br>• Screening<br>• "Technology-based dietary assessment"<br>• Food*record*<br>• Recording<br>• Assessment<br>• Food diary	• Device<br>• Informatics<br>• Technology<br>• Computer<br>• "web-based"<br>• Image*based<br>• Image retrieval<br>• Picture<br>• Digital photography<br>• Mobile device*<br>• Mobile*technology<br>• Smartphone<br>• Technology*assist*<br>• Multimedia tool<br>• Electronic*<br>• Wearable	• Hospital<br>• Primary*care<br>• Care<br>•
**MeSH**	• Food habits<br>• Eating<br>• Diet records<br>• Nutritional assessment		• Signal processing Computer/assisted-instrumentation<br>• Software<br>• Wireless technology/instrumentation	

Table 2. Search terms used for Scopus Search Strategy

Concepts	Nutrition	Monitoring	Tools (systems)	Setting
**Author keywords**	• Nutrition<br>• Malnutrition<br>• Nutritional<br>• Hospital malnutrition<br>• Dietary assessment<br>• Food habits<br>• Eating<br>• Diet records<br>• Nutritional assessment<br>• Nutrition support	• Monitoring<br>• Screening<br>• "Technology-based dietary assessment"<br>• Food*record*<br>• Recording<br>• Assessment<br>• Food diary	• Device<br>• Informatics<br>• Technology<br>• Computer<br>• "web-based"<br>• Image*based<br>• Image retrieval<br>• Picture<br>• Digital photography<br>• Mobile device*<br>• Mobile*technology<br>• Smartphone<br>• Technology*assist*<br>• Multimedia tool<br>• Electronic*<br>• Wearable	• Hospital<br>• Primary*care<br>• Care

Table 3. Search terms used for CINAHL Search Strategy

Concepts	Nutrition	Monitoring	Tools (systems)	Setting
**Author keywords**	• Nutrition<br>• Malnutrition<br>• Nutritional<br>• Hospital malnutrition<br>• Dietary assessment<br>• Food habits<br>• Eating<br>• Diet records<br>• Nutritional assessment<br>• Nutrition support<br>• Food habits<br>• Eating<br>• Diet records<br>• Nutritional assessment	• Monitoring<br>• Screening<br>• "Technology-based dietary assessment"<br>• Food*record*<br>• Recording<br>• Assessment<br>• Food diary	• Device<br>• Informatics<br>• Technology<br>• Computer<br>• "web-based"<br>• Image*based<br>• Image retrieval<br>• Picture<br>• Digital photography<br>• Mobile device*<br>• Mobile*technology<br>• Smartphone<br>• Technology*assist*<br>• Multimedia tool<br>• Electronic*<br>• Wearable<br>• Signal processing computer/assisted-instrumentation<br>• Software<br>• Wireless technology/instrumentation	• Hospital<br>• Primary*care<br>• Care

Table 4. Initial Relevance Screening Form

Questions	Options	Exclusion	Additional Notes
1. **What type of source is the result?**	- Journal Paper (go to question 2)<br>- Conference Paper (go to question 2)<br>- Book Section (go to question 2)<br>- Editorial (go to question 2)<br>- Unpublished work (go to question 2)<br>- "Others" (e.g. Commentary, press article)	- If category is in "others"	
2. **Is an abstract available?**	- Yes (go to question 4)<br>- No (go to question 3)		
3. **Can it be concluded from the title that the article deals with technologically-driven nutrition monitoring?**	- Yes (go to question 4)<br>- No	- If category is in "No"	
4. **Does the article imply that technology is implicated in nutrition monitoring?**	- Yes (go to question 5)<br>- No	- No	
5. **Does the article deal with healthcare?**	- Yes (article will stay for further review process)<br>- No	- No	- Healthcare includes: Prevention of diseases, Patient care, healthcare research, healthcare management

List of Abbreviations

PHAC-Public Health Agency of Canada

CVD-Cardiovascular disease

ADL-Activities of daily living

LOS-Length-of-stay

PFP-Pay-for-performance

ICU-Intensive care unit

CCU-Critical care unit
