# Supplementary material for: The Use of Technology in Identifying Hospital Malnutrition: Scoping Review
Source: JMIR Med Inform. 2018 Jan 19;6(1):e4. doi: 10.2196/medinform.7601 (PMC5797288; doi:10.2196/medinform.7601)
Supplement: Multimedia Appendix 2 [file medinform_v6i1e4_app2.pdf]

Table 1: Adapted Data Characterization Form

Table 1 has been adapted to the original Data Synthesis and Characterization excel spreadsheet used to analyze and categorize the search results.

Authors	Year of Publication	Study Type	Techno-logy type	Aims, Purpose and Measure	Outcomes			
					Time Savings (Y, N)	Cost Savings (Y, N)	Increased Malnutrition Detection/awareness (Y, N)	Accuracy (Y, N)
Zhang	2014	Semi-experimental study	Software	To develop a quantitative assessment tool that can assess whether an individual is malnourished. This is a malnutrition assessment tool.	Not specified.	Not specified.	Not specified, but future potential suggested.	Yes. The authors state Bioelectrical impedance (BIA) and Phase angle (PhA) successfully identified malnourished patients amongst healthy patients. Authors suggest that using PhA can be a quantitative index in assessing nutrition state while other frequently used methods, such as the Subjective Global Assessment (SGA) contain many subjective factors.
Rich	1981	Retrospective study	Hardware	To examine the effectiveness and efficiency of using a portable calculator system for monitoring food intake. This is a nutrition monitoring tool.	Yes. The author suggests that the programmable calculator system has the ability to decrease dietetic workload for a Nutritional Support Service overseeing 20 patients by 10 hours per day (1981) through improved charting time.	Yes. The author compares the cost of the programmable calculator system ($280, January 1981) to larger more sophisticated micro-processing systems ranging in several thousands of dollars.	Yes. The author suggests that the programmable calculator system can quickly identify cases of malnutrition and subsequently address them faster relative to the manual monitoring systems available in 1981.	Yes. The author states that the tool is accurate, however the system is not impermeable to human errors such as measuring quantities and approximations.
Fraser & Turney	1990	Semi-experimental study	Both	To examine if a nutrition expert system can make expert recommendations in the nutrition plan of parenteral patients. This is both a malnutrition assessment and nutrition monitoring tool.	Yes. Although the primary advantage of this system, NUTRITIONAL ADVISOR (NA), is to serve as a decisional support tool for health practitioners.	Not specified.	Not specified.	Not specified. During the current study differences between NA and a nutritional expert, nutritional support recommendations were noted. The authors state that they will improve NA in the next phase of this project.
Slee, Birch, & Stokoe	2015	Semi-experimental study	Software	The aim was to investigate how BIVA and PhA assessments compare to traditional malnutrition assessments, MUST and MNA-SF. This is a malnutrition assessment tool.	Not specified.	Not specified.	Not specified.	Yes. The authors show that Bioelectrical impedance vector analysis (BIVA) and Phase angle (PhA) assessment of malnourished patient data points correlate with MNA-SF classification of malnutrition or at-risk of malnutrition, while the data points with MUST predominantly correlate with patients classified at-low risk for malnutrition.

Wieskotten, Heinke, Wabel, Moissl, Becker, Pirlich, Keymling, Isermann	2008	Semi-experimental study	Software	The aim was to develop a novel (nutrition) assistance system that supports the physician with nutritional assessment, easily. So the authors developed an assistance system using fuzzy logic, and another system using a decision tree and compared both to the malnutrition assessment tool, SGA. This is a malnutrition assessment tool.	Not specified.	Not specified.	Not specified. The authors however do state that the current study provides a direction for future research to quantify the measurement of malnutrition.	Yes. The authors show an 80% accuracy rate in correctly classifying patients as malnourished compared to the SGA assessment. The authors further add that a score of 100% is not attainable compared to the SGA because its nutrition scores partly rely on subjective assessments, and different investigators can obtain different results on the same patient.
Vanderveen & Groves	1986	Semi-experimental study	Software	Aim was to develop a computer assistance tool that has an automated nutritional assessment program to aid in identifying whether patients are malnourished. This is a malnutrition assessment tool.	Yes. The program eliminates the need for manual transcription and the authors believe this will save time.	Not specified.	Yes. The authors state that an advantage of the current nutritional support service (NSS) is rapid and effective nutritional assessment which can facilitate rapid nutritional support.	Not specified. The NSS was not tested on patients in the current study.
Chen, Hsu, Liu, & Yang	2012	Semi-experimental study	Software (Cloud-based)	To develop an expert nutrition diagnosis system using AI to make the nutrition diagnostic process faster and more accurate for the dietician. This is a malnutrition assessment tool.	Yes. The authors tested and compared the total time-spent on making a nutritional diagnosis between their nutritional expert system against an experienced dietician. The results show that the nutritional expert system was faster (1.3 seconds) than the experienced dietician (1.96 seconds) at providing a nutritional diagnosis.	Not specified.	Yes. The authors state that their expert system was able to correctly diagnose all 100 patients in the study, while the experienced dietician misdiagnosed three of those patients.	Yes. The authors' expert system was more accurate at diagnosing a nutritional problem compared to an experienced dietician. The authors also state that the expert system should be used as an aid for healthcare workers to facilitate more accurate and timely nutritional diagnoses.
Ulibarri, Gonzalez-Madrono, Villar, Gonzalez, Gonzalez, Mancha, Rodriguez, Fernandez	2005	Semi-experimental study	Software	To develop a screening tool (CONUT) that allows an automatic daily assessment of nutritional status in all patients for the early detection/continuous control of hospital undernutrition comparable to subjective tools such as SGA & FNA. This is a malnutrition assessment tool.	Yes. The authors state that CONUT is quicker than the Full Nutritional Assessment (FNA), and Subjective Global Assessment (SGA).	Yes. The authors state that CONUT is less costly than the FNA and SGA.	Yes. The authors state that the agreement levels between CONUT and the SGA and FNA are very acceptable in routine clinical examinations. Further they state that CONUT is a valid screening method that can aid in early detection of hospital malnutrition.	Yes. The authors report a high sensitivity (92.3%) and specificity (85%) of CONUT relative to the "gold-standard" nutritional assessment currently in use by some hospitals to diagnose malnutrition, the FNA.
Homar, Blanco, Hernandez, Cortes & Sotelo	2015	Semi-experimental study	Software	Aim is to develop an integrated computer software application for specialized nutritional support, integrated in the electronic clinical record, which both automatically and early detects undernourished patients or patients at-risk of developing undernourishment, determining points of opportunity for improvement and evaluation of the results. This is a malnutrition assessment tool.	Not specified.	Not specified.	Not specified.	Yes. The computer software application employs the automated nutritional screening system FILNUT. For this developed application, the authors state that FILNUT presented a 94.1% positive predictive value, and a 0.83 concordance (kappa index versus a current gold-standard nutritional assessment tool).

Sun, Burke, Mao, Chen, Chen, Bai, Li, Li & Jia	2014	Semi-experimental study	Hardware	Aim is to develop a wearable device that monitors diet, physical activity, guiding the blind, and monitoring those with dementia. The focus here will be on the food intake monitoring function. This is a nutrition intake monitoring tool.	Not specified.	Not specified.	Not Specified.	Yes. The authors evaluated and compared the accuracy of eButton to a physical measurement (ground truth) on food items. After testing 100 common foods, eButton was able to estimate within a 30% margin of error in the majority of cases (85/100). The authors report that this is a significant improvement from their visual estimation of the same foods.
Macdonald, Teal, Bamford & Moynihan	2012	Descriptive study	Both	Aim is to develop an innovative prototype/concept for an improved food service. The concept includes all aspects concerning hospital nutrition, such as tools to facilitate eating, the environment, and tools to facilitate communication about food. This is a nutrition intake monitoring tool.	Not specified.	Not specified.	Not specified.	Not specified.
Giovanelli, Coevoet, Vasseur, Gheysens, Basse & Houyengah	2015	Semi-experimental study	Software	Aim is to develop a software algorithm that can detect readmitted patients with malnutrition during a previous stay. An email would be sent to the nutrition department, saving time spent on doing nutrition assessments. This is a malnutrition assessment tool.	Not specified. The authors state that the tool was designed to facilitate quicker provisions of nutritional care.	Yes. The authors state that the tool has improved the financial impact of stays for malnourished patients on the hospital budget.	Yes. The tool has the capability to automatically alert the nutrition department about a previously admitted malnourished patient.	Yes. The authors calculated a positive predictive value (PPV) (88.9%) for the tool, which corresponds to the probability that the patient was malnourished when an alert was sent.
Rossi, Campbell, & Ferguson	2013	Semi-experimental study	Software	The aim was to compare the efficiency and effectiveness of an electronic based nutrition care system to a conventional paper-based nutrition care system. This is a malnutrition assessment tool.	Yes. The authors state that the dietician was able to reduce the total time spent completing a full nutritional assessment by 13 minutes by using the electronic system (57 minutes/patient) compared to the paper-based system (70 minutes/patient).	Yes. The authors estimate an annual savings of USD$6,500-$10,000 through a time savings in dietician workload, assuming an hourly rate of $25/h with 260-390 hours saved.	Yes. The authors state that a greater number of nutrition related diagnoses were resolved over a 6 month period using the electronic system compared to the paper-based system.	Not specified.
Brieux, Kaminker, Campos, Guillen, Alejandris, Luna, Baum, & de Quiros	2014	Randomized Control Trial	Software	The aim of the study is to assess the outcomes of a nutritional-risk alert (malnutrition) by using an informatics system in hospitalized patients versus the conventional method. The objective is to prove that an alert is a helpful tool for doctors to better detect malnourishment in hospitals. This is a malnutrition assessment tool.	Not specified.	Not specified.	Yes. The informatics system alert in the experimental group detected more cases of malnutrition (34.3%) when compared with an absence of the informatics system in the control group (20.3%).	Yes. The authors report that the informatics system alert had high sensitivity (0.99) and high specificity (0.98) compared to their gold standard. The study's gold standard was created by a committee of experts that retrospectively analysed all of the medical record information to determine whether patients of either the control or experimental groups were clinically malnourished.
Krenning	1983	Descriptive study	Hardware	The study aims to describe a pocket computer that allows for versatile and rapid nutritional evaluation for hospitalised patients. This is a malnutrition assessment tool.	Not specified.	Not specified.	Not specified.	Not specified. The author states that the computer program includes standard nutritional formulae with the addition of an ideal body weight formula derived specifically for this system.

McGurk, Jackson, & Elia	2013	Semi-experimental study	Software	The study aimed to examine the efficacy, reliability, feasibility, efficiency and user-friendliness of a electronic nutritional self-screening procedure. More importantly it sought to examine the agreement between self-screening and healthcare professional screening results for possible consideration of routine implementation of the procedure. This is a malnutrition assessment tool.	Yes. The authors state that self-screening tools can be a rapid and viable option to reduce the workload of healthcare workers.	Not specified.	Not specified.	Yes. The authors state that there was perfect agreement between self-screening and healthcare worker re-screening (kappa = 1.00).
Comber, Weeden, Hoare, Lindsay, Teal, Macdonald, Methven, Moynihan, & Olivier	2012	Semi-experimental study	Both	The study aimed to describe their design and validation of a computer-assisted tool for the visual assessment of nutrient intake of patients. This is a nutrition intake monitoring tool.	Not specified. The authors do state that the application could prove beneficial in providing timely care to malnourished patients and could decrease the efforts required to monitor nutrition by healthcare workers.	Not specified. The authors do state that the application could reduce hospital costs related to nutrition monitoring.	Not specified. The authors state that the application could improve patient monitoring.	Yes. The authors state that the average weight scores of estimated food consumption compared to the actual weight of the food were on average underestimated by the users of the application by 5 grams. Intra-rater reliability coefficient was 0.916 (5 ratings) and 0.954 (6 ratings). Inter-rater reliability coefficient was 0.856.
Visser, Venrooij, Wanders, Vos, Wisselink, Leeuwen, & Mol	2012	Semi-experimental study	Hardware	The study aimed to investigate whether Bioelectric impedance Phase Angle can be used as a rapid indicator of undernutrition in cardiac surgery patients. This is a malnutrition assessment tool.	Not specified.	Not specified.	Not specified.	No. The authors state that there are significant associations between PA and undernutrition, however there is no agreement about the accuracy of PA as an indicator of undernutrition. The authors recommend further research within a clinical trial to determine if this association is causal.
Llido	2006	Semi-experimental study	Software	The aim was to introduce a hospital nutrition support program to improve hospital health care delivery by identifying malnutrition in all admitted patients and following up those identified to be malnourished and ‘‘at risk of developing malnutrition’’. This is a malnutrition assessment tool.	Not specified. The author states that the computer program results in faster data archiving and retrieval, interpretation and reporting, allowing for a more timely and up-to-date patient status and leading to better patient-care delivery.	Not specified.	Yes. The author states that referrals to the nutrition support team based on screen notifications increased from 37% to 100%. Entry of patient data (height and weight) improved from 30% to 90% by healthcare workers. Medical staff increased their referrals to clinical dieticians from 38% to 83%.	Not specified. The author states that the nutrition screening and assessment portion for determining the BMI-based nutritional status for patients were based on the values recommended by the World Health Organization.
Sullivan, Bopp, Robertson, Lensing & Sullivan	2016	Semi-Experimental study	Both	The aim was to test the effectiveness and reliability of the multi-component method (MCM) for recording nutrient intake in the hospital setting. The multi-component method is a computer system that uses photography, observation and a Window’s Access computer program.	Yes. The authors state that the MCM rendered a time savings of 67% compared to the traditional method used by hospital dietary staff.	Not specified.	Not specified. The authors state that real-time results are available to the clinician, who can then respond to a problem in a timely manner.	Yes. The authors report that the MCM is an accurate and reliable method for estimating nutrient and calorie intakes. (Intraclass correlation coefficient = 0.975, 95% CI 0.958-0.992)

Hershkovich, Stark, Levi, Weiner, Gur, Rozen	2016	Descriptive Study	Software	The aim of the study was to support the universal use of automated tools, such as the Rambam Automated Nutrition Computerized Screening (RANCS) tool, to nutritionally screen all admitted patients for the risk of malnutrition.	Yes. The authors state that since RANCS updates automatically with certain laboratory biomarkers, no additional time is spent recording information.	Yes. The authors state that RANCS is able to reduce hospital costs, although do not specify the amount.	Yes. The authors compared screening patients for malnutrition using the Malnutrition Universal Screening Tool (MUST) with RANCS. The time spent using MUST was 12.2+/-5.3 mins, while no additional time was spent for RANCS.	Yes. The authors report that RANCS is accurate. Both RANCS and MUST were able to identify 13 high risk patients from a group of 94. Sensitivity and specificity of RANCS to MUST was 72.2% and 78.9%, respectively.
